# Skin manifestations associated with COVID-19^☆^

**DOI:** 10.1016/j.abd.2021.08.002

**Published:** 2021-11-10

**Authors:** Camila Arai Seque, Milvia Maria Simões e Silva Enokihara, Adriana Maria Porro, Jane Tomimori

**Affiliations:** Department of Dermatology, Universidade Federal de São Paulo, São Paulo, SP, Brazil

**Keywords:** COVID-19, Dermatology, Skin

## Abstract

This article will address the main aspects of skin manifestations associated with COVID-19, based on a review of the literature published to date. Since the beginning of the pandemic, more than 1,500 articles have been published on the subject. Regarding the pathophysiology, it is believed that the same mechanisms responsible for the disease in the main target organs also act in the skin, although they are not yet fully elucidated. The actual frequency of dermatological manifestations remains uncertain – it can range from 0.2% to 45%, being close to 6% in systematic reviews. Pioneering studies of large case series conducted in European countries and the USA provide the first information on the main skin manifestations associated with COVID-19 and propose classifications regarding their clinical presentation, pathophysiology, as well as their frequencies. Although there is yet no consensus, maculopapular eruptions are considered the most frequent presentations, followed by erythema pernio-like (EPL) lesions. Manifestations such as urticaria, vesicular conditions and livedo/purpura/necrosis are rare. The time of onset, severity, need for specific treatment and prognosis vary according to the clinical presentation pattern. The increasing histopathological description of skin conditions can contribute to the diagnosis, as well as to the understanding of the pathophysiology. Also, in the dermatological field, the relationship between COVID-19 and androgens has been increasingly studied. Despite all the generated knowledge, the actual biological meaning of skin manifestations remains uncertain. Therefore, the exclusion of the main differential diagnoses is essential for the correlation between skin manifestation and COVID-19.

## Introduction

At the beginning of the COVID-19 pandemic, skin lesions weree already considered to be important among the clinical manifestations of this new disease. Since March 2020, more than 1,500 articles correlating “dermatology” and “COVID-19” have been published. Never has such a large amount of information been generated in such a short time in the scientific community, including dermatology-related data. Although the first reports were poorly informative, possibly due to the inability to perform a complete dermatological examination, photographic record, histopathological and/or immunohistochemical analysis, and the scarcity of specialists in direct assistance to these patients, subsequent research has produced important contributions on the cutaneous involvement in COVID-19.^1^ In this document, the main publications to date (04/30/21) on different aspects of the dermatological manifestations of COVID-19 will be reviewed, according to the methodologies adopted in these studies. Pubmed was used as a database, using the following keywords: COVID-19, SARS-CoV-2, “dermatology”, “skin”. The initial information is shown in Table 1 (Capsule with the main key points).

**Table 1:** Capsule with the main key points.

Key points
The first report in the literature of a skin manifestation associated with COVID-19 occurred on 03/23/20. Since then, there have been more than 1,500 publications on the subject.
The frequency of skin manifestations ranges from 0.2% to 45%, being close to 6% in systematic review studies
The most frequently described manifestations are maculopapular eruptions, followed by the erythema pernio-like (EPL) lesions
Urticarial, vesicular and the livedo-purpura-necrosis spectra are less frequent
Skin lesions usually accompany the general symptoms. However, urticaria and vesicles may precede them, while EPL lesions and livedo-purpura-necrosis appear later.
The main differential diagnoses are drug reactions and other viral infections, which should be ruled out.

## Frequency of cutaneous manifestations associated with COVID-19

Although the first report in the literature of a dermatological condition possibly associated with COVID-19 was published on 03/22/20 by Joob and Wiwanitkit^2^ in the Journal of the American Academy of Dermatology (JAAD), in late 2019, Guan et al.^3^ made the first citation about the frequency of cutaneous manifestations. Of the 1,099 described patients with SARS-CoV-2 infection, 0.2% had a rash.

The first article focusing mainly on the frequency of cutaneous manifestations, published by Recalcati^4^ on 03/26/20, reported that of 88 cases evaluated by dermatologists, 20.4% had skin lesions. Since then, the estimation of such frequency has been widely variable, in different countries, according to data obtained through different methodologies. Matar et al.^5^ in France, Reknimitr et al.^6^ in Thailand, and Solak et al.^7^ in Turkey have shown frequencies of 1% (n = 756), 15% (n = 183), and 18.3% (n = 382), respectively, through remote collection of data through telephone interviews after evaluation performed by the patient (self-examination). Lower frequencies were recorded by Gaspari et al.^8^ in Italy (1.4%), Tammaro et al.^9^ also in Italy (1.5%, n = 130), and Tamai et al.^10^ in Japan (4.3%, n = 69) through in-person evaluation of patients performed by a dermatologist, a crucial factor for greater sensitivity in detecting skin lesions and accuracy in attributing them to COVID-19. Using the most appropriate methodology, it is believed that the actual frequency is close to those reported by these latest studies. The main criticisms of the initial studies are remote evaluation, assessment by the patients themselves or by health professionals who were not dermatologists, the inclusion of suspected cases of COVID-19 (unconfirmed), and not excluding common skin conditions, such as other viral infections and adverse drug reactions. The actual biological meaning of these manifestations still seems uncertain. More data on frequency will be cited below in the topics “Prospective studies” and “Systematic reviews” and summarized in Table 2 (frequency of dermatological manifestations in COVID-19).

**Table 2:** Frequency of dermatological manifestations in COVID-19.

Author	Country	Frequency	n	Type of evaluation
Guan et al.^3^	China	0.2%	1099	Not in-person (database)
Recalcati et al.^4^	Italy	20.4%	88	In-person by dermatologist
Matar et al.^5^	France	1%	756	Self-examination by telephone interview
Rerknimitr et al.^6^	Thailand	15%	183	Self-examination by telephone interview
Solak et al.^7^	Turkey	18.3%	382	Self-examination by telephone interview
Gaspari et al.^8^	Italy	1.4%	NA	In-person by dermatologist
Tammaro et al.^9^	Italy	1.5%	130	In-person by dermatologist
Tamai et al.^10^	Japan	4.3%	69	In-person by dermatologist
De Giorgi et al.^31^	Italy/China	7.8%	678	In-person (does not specify evaluator)
Rehktman et al.^32^	USA	11.8%	296	In-person by medical students
Méndez-Maestro et al.^30^	Spain	18.7%	75	In-person by dermatologist
Nuno-Gonzales et al.^34^	Spain	45.65%	666	In-person by dermatologist
Jamshindi et al.^36^	NA^a^	5.95%	1847	NA^a^

NA, Not Available data.

a Meta-analysis study.

Interestingly, a Spanish study showed that the frequency of dermatological manifestations was even lower in the second wave of the epidemic in that country.^11^ Possible explanations are: better knowledge about the disease allowed early treatment and more assertive treatment, which reduced the proportion of severe cases and adverse drug reactions (one of the main differential diagnoses); greater application of preventive measures and diagnostic tests to exclude other viral infections; new mutations with a greater diversity of virulence and antigenicity. Furthermore, the apparent low frequency of dermatological lesions compared to “classic” clinical manifestations can be explained by the lower expression of viral receptors in the skin compared to other target organs and by the presence of membrane proteases with a protective action on the skin.^12^

Finally, a recent Austrian study with case-control methodology did not show any statistical significance comparing the presence of skin lesions in the group of patients with confirmed COVID-19 (cases) and in the non-COVID group (controls).^13^ It is suggested that skin lesions are less specific than other clinical signs, are not good diagnostic clues, should be evaluated carefully, and be attributed to COVID-19 only after ruling out other differential diagnoses, such as adverse drug reactions and other viral infections.

## Pathophysiology

The mechanisms by which SARS-CoV-2 causes disease in different organs, including the skin, are quite complex and not yet fully understood.

The risk for an organ to develop SARS-CoV-2 infection is determined by the presence of functional ACE2 (angiotensin-converting enzyme 2) and TMPRSS2 (transmembrane protease serine 2) viral receptors expressed on local cells. ACE2 is found mainly in alveolar epithelial cells, explaining the high vulnerability of the lungs to COVID-19. Some studies have shown that ACE2 is detectable in the basal layer of the epidermis, as well as in hair follicles. However, the presence of SARS-CoV-2 in skin samples has been related to the expression of ACE2 in cutaneous capillary endothelial cells, not in keratinocytes and melanocytes,^14^ which makes the virus unable to penetrate and infect directly and primarily the skin. Skin involvement is indirect, and it occurs due to infection of the endothelial cells of the dermal vessels. The presence of SARS-CoV-2 in skin lesions has been previously confirmed by the PCR (polymerase chain reaction) technique in tissue with low replication, even in patients with negative PCR in the nasal swab and serology, showing that the skin can be an infected organ, and eventually even a diagnostic one.^15^

The first and strongest evidence about the pathophysiology of skin lesions in COVID-19 was published by Magro et al.^16^ at the beginning of the pandemic (04/15/20). Through histopathology techniques and immunohistochemical analysis of complement components and the viral spike protein, the study showed that the pathophysiological process responsible for the pulmonary involvement can also occur in the skin, both healthy and damaged, in addition to demonstrating the viral presence in these organs. Similarly, Colmenero et al.^17^ also identified viral particles in the endothelium of erythema pernio-like lesions in children, which supports the causal relationship between cutaneous involvement and viral presence. SARS-CoV-2-induced endothelial damage in the skin may be one of the key mechanisms in the pathogenesis.

The pathophysiology of COVID-19 is known to be multifactorial and involves the innate immune, humoral response, hypercoagulability, monocytic/macrophagic activation, significant cytokine increase (“cytokine storm”), among others.^18^ In a rather simplified way, skin manifestations have been divided into two forms regarding the pathophysiology: (1) inflammatory, as an immune response to viral nucleotides, and (2) vascular, secondary to vasculitis, vasculopathy, and thrombosis phenomena.^19^ Regarding the inflammatory response, viral infections are related to the triggering of the innate and adaptive immune responses. The monocytic-macrophage system produces an exacerbated immune response, generating an intense systemic inflammatory process. The viral activation of mast cells and basophils could justify lesions such as urticaria and exanthems.^20^ Regarding the vascular mechanisms, the most often studied lesions are the erythema pernio type, in analogy to the pathophysiology of previously known diseases, such as the “classic” erythema pernio (triggered by cold), “lupus”, and other vascular entities, such as livedoid vasculopathy.^21^ It is believed that erythema pernio associated with COVID-19 also results from an exaggerated immune reaction with signaling via type I interferon, which is important for viral eradication, but with a generalized, highly inflammatory response. In critically ill patients with purpura, vascular thrombosis in the skin and other organs would be associated with a minimal interferon response, allowing excessive viral replication, with the release of viral proteins that are located in the extrapulmonary endothelium and trigger extensive complement activation.^22^, ^23^

Controversies regarding the pathophysiology and the presence of viral particles triggering skin lesions are based on studies that verify the absence of these particles in research, using the PCR technique in biopsies of skin lesions.^24^ Additionally, it has recently been suggested that SARS-CoV-2 could cause the reactivation of other latent viruses, such as HHV-6, which is the actual responsible for some skin manifestations initially attributed to COVID-19,^25^ such as exanthems and pityriasis rosea-like conditions.

## Large national and international series

Soon after the first reports of isolated cases of cutaneous manifestations in patients with COVID-19, especially with the arrival of the pandemic in Europe, some countries carried out studies with national and international collaboration under the leadership of their respective academic societies. Thus, the first studies of large series of cases on the most prevalent skin manifestations were reported, including suggestions for classification into groups according to the clinical pattern and pathophysiological mechanisms. These studies were crucial for the initial understanding of the relationship between dermatology and COVID-19 and are still important references to date. The main data from these studies are shown in Table 3 (Large national and international series on skin manifestations).

**Table 3:** Large national and international series on skin manifestations.

Author	Country	n	Confirmed cases	Maculopapular eruption	Urticarial	EPL	Vesiculobullous	Livedo-purpura-necrosis
Galvan-Casas et al.^26^	Spain	375	234 (62,4%)	47%	19%	19%	9%	6%
Masson et al.^27^	France	277	25 (9%)	9%	9%	75%	15%	4%
Freeman et al.^28^	USA +31 countries	716	171 (23,8%)	45%^a^	16%	18%	11%	6,4%
Manzano et al.^29^	Italy	200	120 (62%)	25,7%	10,2%	24,6%	15,5%	9%^b^
Visconti et al.^30^	United Kingdom	11544	694 (6%)	41,2%^c^	30%	23,1%	c	–

EPL, Erythema pernio-like lesions.

a Include morbilliform eruption, maculopapular and papular squamous erythema.

b Include purpura/vasculitis, livedo reticularis/racemosa.

c Include erythematous-papular and erythematous-vesicular eruptions.

### Spain

The first large retrospective series was published on 04/29/20 by Galván-Casas et al,^26^ widely publicized, containing a dermatological atlas. With support from the Spanish Academy of Dermatology, dermatologists across the country included images of skin lesions from 375 patients with COVID-19 (suspected or confirmed), which were grouped into five main patterns: maculopapular eruptions (47%), urticarial rash (19%), EPL (19%), vesico-bullous (9%), and livedo/necrosis (6%).

The most commonly found pattern was maculopapular eruptions, showing a concomitant onset with general symptoms and an average duration of 8.6 days, similarly to urticarial conditions. Both were considered not useful for the diagnosis of COVID-19 because they are non-specific manifestations common to other common conditions, such as adverse drug reactions and other viral infections.

EPL lesions occurred in younger patients with mild systemic conditions, with a late-onset (mean of 12.7 days from the onset of general symptoms) and prolonged duration. Only one of the 71 cases reported prior erythema pernio (EP). They would be useful as an epidemiological and not a diagnostic marker.

Vesicular lesions appeared before the general symptoms in 15% of the cases in this series, with a mean duration of 10.4 days and relative diagnostic specificity since they are skin manifestations not reported in viral exanthems.

Livedo/necrosis includes a variable range of presentations related to COVID-19 or its complications, such as a hypercoagulable state and vascular damage. They are associated with an older age group and higher mortality.

Although there is great merit regarding the pioneering and significant number of cases, the study limitations are the inclusion of patients who were not confirmed cases of COVID-19 (suspected cases), mostly outpatients, evaluated photographic records, without histopathological analysis or exclusion of the main differential diagnoses.

### France

On 05/04/20, de Masson et al.,^27^ with support from the French Union of Dermatologists and Venereologists, published a retrospective observational study in letter form that included images of skin manifestations of 277 patients with COVID-19 (252 suspected and 25 confirmed cases) from private clinics throughout France. Surprisingly, EPL lesions corresponded to the vast majority of cases (75%) and were even reported as the single manifestation of the disease. Histopathology study carried out in three cases showed lichenoid interface dermatitis with the presence of microthrombi. Other identified cutaneous patterns were: vesicular (15%), urticarial (9%), morbilliform (9%), petechial (3%), livedo reticularis (1%), and others (15%). Some patients were accounted for in more than one category. It is worth remembering that the French were the first to describe EPL lesions related to COVID-19, which could be a bias for the high frequency shown in this study, non-reproducible in other similar studies. Additionally, the high prevalence of EPL lesions is probably reflected in the large percentage of unconfirmed cases included in the analysis since such lesions predominate in oligo- or asymptomatic COVID-19 cases, which are seldom tested for laboratory confirmation of SARS-CoV-2 infection.

### USA/International (31 countries)

In collaboration with the American Academy of Dermatology and the International League of Dermatological Societies, Freeman et al.^28^ collected data on 716 cases from 31 countries through an electronic registry and the results were published on 07/02/20. Dermatologists provided data on 54% of the cases, with 89% of all registries originating from the USA. Only 34 Hispanic/Latino and 13 African-American patients were included, impairing the racial diversity required to understand the skin manifestations. Only 171 patients were confirmed COVID-19 cases, with the following frequency of manifestations: morbilliform (22%), EPL (18%), urticarial (16%), macular erythema (13%), vesicular (11%), papulo-desquamative (9.9%) and retiform purpura (6.4%). Most of the skin manifestations started concomitantly with the general symptoms; however, in 12%, the skin lesions were the first symptoms of the disease, showing the possible importance of skin lesions as a marker for the early diagnosis of COVID-19. Regarding the severity, EPL lesions were associated with milder systemic conditions, while 100% of cases with retiform purpura were hospitalized. Histopathology was carried out in 14 confirmed cases. The study limitations include evaluator bias, the impossibility of estimating the frequency/prevalence of skin manifestations, low rate of confirmed COVID-19 cases (25%), and little ethnic diversity, despite the apparent high representativeness of the 31 countries.

### Italy

Similar to Spain and France, on 01/18/21, the Italian Society of Dermatology and Sexually Transmitted Disease also coordinated a national study that gathered data from 200 patients with skin manifestations. One of the inclusion criteria for Marzano et al.^29^ was the presence of COVID-19-related skin lesions confirmed by an experienced dermatologist. A total of 62% had a confirmed diagnosis of COVID-19. The main identified patterns were: maculopapular eruption/morbilliform exanthem (25.7%), EPL (24.6%), papulovesicular eruption (15.5%), urticarial eruption (10.2%), purpura/vasculitis (6.9%), livedo reticularis/racemosa (2.1%) and others (15%), which included pityriasis rosea-like, erythema multiforme-like, erythema nodosum-like, panniculitis and angioedema. Mucosal lesions were not identified. The skin condition appeared on average 14 days after the onset of systemic symptoms and lasted for an average of 12 days. EPL lesions lasted longer than the other patterns, occurred in a younger age group, and represented a lower risk of developing a severe systemic disease, all factors with statistical significance. The mean age of the livedo reticularis/racemosa and maculopapular eruption/morbilliform exanthem groups was also older (p < 0.01). One limitation of the study is the lack of diagnostic confirmation of SARS-CoV-2 infection in 36.5% of the sample due to economic factors that limited the extensive diagnostic testing in Italy and in several other countries.

### United Kingdom

Finally, the most recent population study, with very significant figures, whose results generated a lot of repercussions, was published on 01/14/21 by Visconti et al.,^30^ which included data from all over the United Kingdom and had a different design than those of the abovementioned publications.

The first arm of the study collected data from 336,847 users of the “COVID Symptom Study” application, in which they provided epidemiological, laboratory, and clinical information, including on the presence or absence of skin lesions identified by the users themselves. Only 2021 (7.4%) users had laboratory confirmation of COVID-19, and of these, 178 (8.8%) reported skin lesions. The prevalence was slightly higher among women. Additionally, the prevalence of skin lesions was significantly higher among those who tested positive for SARS-CoV-2 when compared to those who tested negative, justifying the inclusion of skin involvement in the list of symptoms in suspected COVID-19 infection due to its diagnostic, predictive factor, regardless of how often it occurs.

The second arm of the study sought to investigate in detail the skin manifestations through a questionnaire addressed to users who reported them and the sharing of photos. Of the 11,544 answered questionnaires, only 694 (6%) had a confirmed diagnosis of COVID-19. In this group, although the majority reported the onset of skin lesions together or soon after the general symptoms, 17% reported that the skin lesions appeared before the systemic symptoms, and in 21%, the skin condition was the only symptom. However, the study did not provide additional detailed information about these findings. There was no description of which skin manifestations occurred prior to the general symptoms and how long before they appeared. Neither did it specify which skin lesions were considered by the individual themselves as the only manifestation of COVID-19, nor did it clarify the reason why laboratory confirmation of the disease was performed in the absence of any suspected symptoms.

In this same arm of the study, 2,328 users shared photos of skin lesions; 260 images were randomly selected for analysis by dermatologists, and of these, 30 were discarded as not attributable to SARS-CoV-2 infection – examples: acne, molluscum contagiosum, herpes zoster, eczema, perioral dermatitis, impetigo, dermatophytosis, which shows the user's own limitations as an evaluator of their skin lesions. The final images were evaluated by four experienced dermatologists. The most frequent dermatological presentations were papular eruptions (41.2% including erythematous-papular and erythematous-vesicular), urticarial (30%), and acral (23.1%) eruptions.

To date, this is the first and only impactful publication stating the frequency and relevance of skin lesions as the only manifestation of SARS-CoV-2 infection. Therefore, caution and further studies on the subject are necessary to understand its actual importance and impact on the diagnosis of COVID-19.

The nature of the study has four main limitations: data based on self-assessment, not representative of the general population, disregarding of rare dermatological manifestations, and the impossibility of ruling out important differential diagnoses, such as adverse drug reactions.

## Prospective studies

There are few prospective studies on skin manifestations associated with COVID-19 that have been published to date. Such methodology would provide more reliable information about the actual prevalence of skin lesions and the identified patterns, especially through the active search for skin lesions by the specialist.

Nevertheless, different prevalence rates, proportions, and classifications of skin manifestations diagnosed by dermatologists in patients with prospectively confirmed SARS-CoV-2 infection have been reported. De Giorgi et al.^31^ evaluated 678 patients in hospitals in Italy and China, finding a frequency of 7.8% of skin lesions, with 44% being identified at the diagnosis of COVID-19 and 56% in subsequent days, on average after 11.7 days. The most frequent finding was exanthem (70%), followed by urticarial (26%) and vesicular lesions (4%). All exanthem cases spontaneously improved within two to five days. Rehktman et al.^32^ evaluated 296 patients in two hospitals in New York, with an 11.8% frequency of skin lesions in the following proportions: ulcers (37.1%), purpura (25.7%), necrosis (14.3%), non-specific erythema (11.4%), morbilliform eruption (11.4%), EPL (11.4%) and vesicles (2.9%). Méndez-Maestro et al.^33^ evaluated 75 patients in a Spanish hospital, and 14 (18.7%) had skin lesions that were possibly associated with COVID-19, namely: EPL (42.8%), maculopapular eruption (28.6%), urticarial (14.3%), livedo reticularis-like (7.15%) and vesicular lesions (7.15%). Finally, Nuno-Gonzales et al.^34^ evaluated 666 patients, also in a hospital environment in Spain, and reported a very high frequency of 45.65% of mucocutaneous lesions. However, the study included very non-specific changes that were both mucosal (examples: papillitis, glossitis, stomatitis, depapillation, mucositis) and cutaneous (examples: erythematous-brownish palmoplantar macules).

In Brazil, there are no data so far on the prevalence of skin lesions. Avancini et al.^35^ reported that of the 86 patients admitted to a referral center with a confirmed infection by SARS-CoV-2 for which an evaluation by the dermatology team was requested, it was not possible to attribute a direct correlation between skin lesions and COVID-19 in any of them, since the identified profile of dermatoses was similar to that of the pre-pandemic period. Five patients had exanthem that was indistinguishable from a drug reaction, and one had EPL lesions, but together with a personal history of systemic lupus erythematosus.

## Systematic reviews

A large number of publications in a short period of time allowed the first systematic review of studies on the skin manifestations of COVID-19 to appear at the end of 2020, aiming to gather and compile the results obtained up to that time, producing data with greater scientific evidence. Still, the conclusions were divergent.

In two systematic review articles, the prevalence of skin lesions in patients with COVID-19 was quite similar: 5.69 and 5.95%.^36^ However, even meta-analysis studies still differ as to what would be the most frequent skin manifestations. Most systematic reviews found a higher frequency of maculopapular eruptions: 37.1%, 36% and 48%, followed by vascular lesions (including EPL): 23% and 33%.^37^ However, others claim that the most common pattern would be EPL lesions (51.5%).^38^ Urticarial, vesicular, and livedo/necrosis conditions are considered rarer.^36^, ^37^, ^38^

In their systematic review, Jamshidi et al.^36^ focused on prognostic factors associated with different patterns of skin manifestations. Maculopapular eruptions are associated with mild COVID-19 in 48% of cases, as well as urticarial and EPL patterns. Patients with vascular lesions of the livedo, purpura and necrosis spectrum were associated with the highest mortality (18.2%), and those with urticarial lesions, the lowest (2.2%). Regarding the time of onset of skin manifestations, the vast majority (92%) occur within four weeks of the onset of COVID-19 general symptoms, especially in the first days and up to two weeks in cases of exanthems and urticaria. On the other hand, EPL lesions occur later and appear between the second and fourth weeks, as well as purpura and necrosis. In 8 to 10%^37^ of the cases, the skin manifestations may precede the clinical symptoms, on average, by three days, thus possibly being useful indicators for the early diagnosis of COVID-19. It is important to emphasize that almost all the studies included in the systematic reviews originate from Europe and the USA, with few reports from Asia and Latin America (due to the scarcity of published data), which could generate a bias.

## Main skin manifestations associated with COVID-19

### Maculopapular eruption

**Figure 1:**
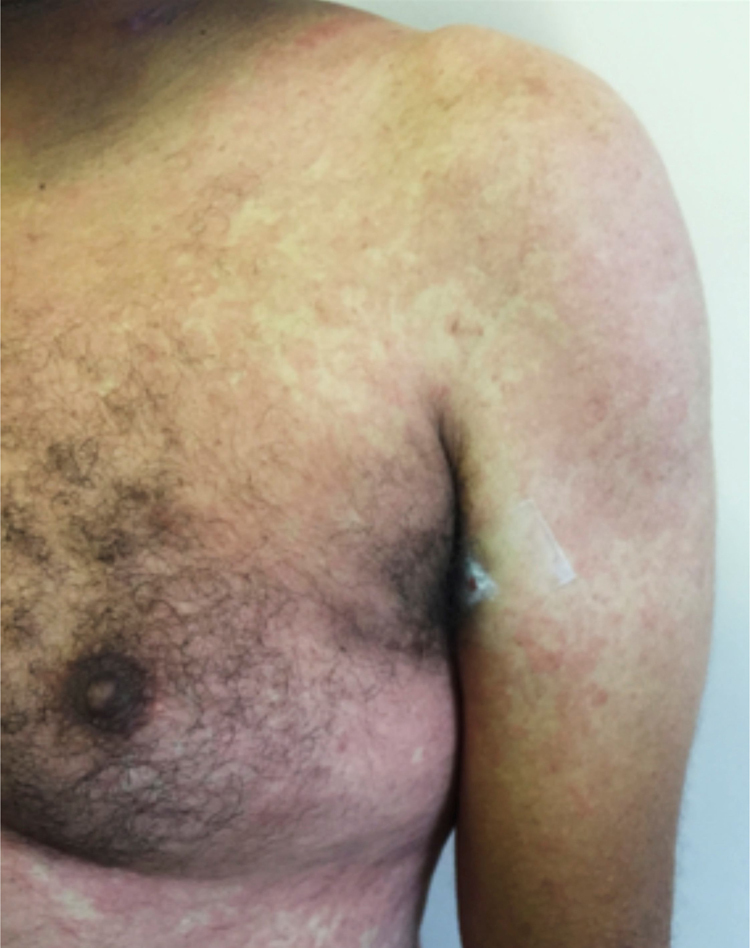
Exanthem in a patient with COVID-19.

In most studies, it is considered the most frequent skin manifestation of COVID-19. It includes exanthem (Fig. 1), rash, diffuse papules, pityriasis rosea-like, erythema multiforme-like, among others. It usually appears within the first days of the general viral infection symptoms or in up to two weeks, although there are sporadic reports of a late-onset (after one month). It correlates with mild systemic conditions and spontaneously improves within seven to ten days,^26^ without the need for specific treatment.

Although these are frequent manifestations, they are highly non-specific, since other viral infections are common causes of exanthems, as well as adverse drug reactions. The literature discusses the need to exclude such differential diagnoses before attributing the skin condition solely to COVID-19, even in confirmed cases of the disease. Another confounding factor is the viral reactivation that occurs in cases of drug reactions, such as the activation of HHV-6 in DRESS (drug reaction with eosinophilia and systemic symptoms). One hypothesis would be that the SARS-CoV-2 infection would lead to reactivation of latent viruses, such as HHV-6, and thus predispose to an adverse drug reaction.^25^, ^39^

### Erythema pernio-like (EPL)

It is undeniable that this is the most often reported manifestation in large case series in the literature.^40^, ^41^ With the arrival of the COVID-19 pandemic in Europe, there was an explosion of cases similar to “classic” erythema pernio, but with peculiar characteristics, different from the classic form: predominance in children and young individuals, at a time of mild temperatures (spring), with no personal history of erythema pernio, collagenosis or other predisposing conditions. Then, EPL associated with COVID-19 was described. Since then, there have been numerous publications on the subject, including those showing the presence of the viral spike in skin lesions,^17^ even in patients with a negative PCR result (swab). EPL is more frequent in healthy, oligo- or asymptomatic children and young adults with COVID-19, and it may be the only manifestation of the disease. Therefore, in the presence of these lesions, it is suggested that SARS-CoV-2 infection should be investigated. It appears on average after 10 to 14 days of the onset of general symptoms, lasts an average of 14 days, with spontaneous improvement.^41^ Most patients (in some series, up to 100% of them) have a negative PCR (swab) viral test result. This fact could be explained by the low viral load usually found in oligo- or asymptomatic COVID-19, in children, and in the later stages of the disease (10 to 14 days after symptoms onset). On the other hand, the absence of PCR positivity, therefore, of the laboratory confirmation of SARS-CoV-2 infection, raises the question of whether these patients were really infected. Immunohistochemistry and immunofluorescence studies may contribute to elucidating the relationship between EPL and COVID-19.^42^

Recently, the relationship between EPL and SARS-CoV-2 infection has been widely questioned in the literature. One of the main controversies is precisely the lack of proof of SARS-CoV-2 infection in these patients. Case series of EPL have been published, which had no epidemiological, clinical, or laboratory evidence to support the diagnosis of COVID-19,^43^ or even serological evidence, which could contribute to the pathophysiology of EPL.^44^ Although the presence of the viral spike in EPL lesions has already been demonstrated by immunohistochemical techniques, and even the complete viral structure has been identified by electron microscopy, these were specific reports in the literature - the vast majority of publications on EPL do not have this evidence. Regarding the pathophysiology, at the beginning of the pandemic, EPL, livedo, purpura, and necrosis lesions were interpreted as phases of the same spectrum of skin involvement. It is currently believed that they have distinct pathophysiological mechanisms.^45^

### Urticarial lesions

Infections, including viral ones, are known triggering factors for urticarial lesions. In this sense, SARS-CoV-2 infection could be a new triggering factor. Urticarial conditions associated with COVID-19 have been reported in patients without previous reports of the disease. A study in a private clinic reported an increased incidence of urticaria cases diagnosed during the pandemic, but no investigation was carried out regarding the presence of COVID-19 in these patients. Clinically, the lesions are disseminated, indistinguishable from other causes, and angioedema is rare. It is estimated that in 10% of patients, the skin condition may precede the general symptoms of COVID-19 by a few days, and, therefore, the detection of skin lesions could favor the early diagnosis of the infection. However, for the most part, urticarial lesions appear concomitantly with other symptoms. There is no age group predilection, and it is associated with mild systemic conditions with low mortality. Spontaneous improvement is reported within seven days or with the use of antihistamines and/or low-dose systemic corticosteroids.^46^

### Vesico-bullous eruptions

**Figure 2:**
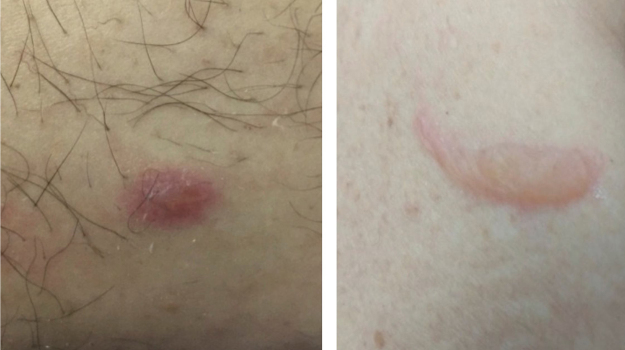
Vesico-bullous lesions in a patient with COVID-19.

Although rare, vesico-bullous eruptions are considered a specific pattern of skin lesion associated with COVID-19, as they are not commonly seen in viral exanthem and drug reactions (main differential diagnoses). They consist of localized or disseminated vesicles or bullae (Fig. 2), which can affect the palmoplantar region, sparing mucous membranes. Their onset occurs approximately three days after the general symptoms and lasts for seven to 14 days, with spontaneous improvement and are associated with a mild to moderate systemic condition. In a series of cases, an attempt was made to isolate the viral agent from the bullous content using the PCR technique, without success. It is crucial to rule out other viral infections, especially herpetic ones, as a case of disseminated herpes with pneumonia has already been reported in a COVID-19 patient, showing the importance of the differential diagnosis.^47^

### Livedo, purpura, necrosis

**Figure 3:**
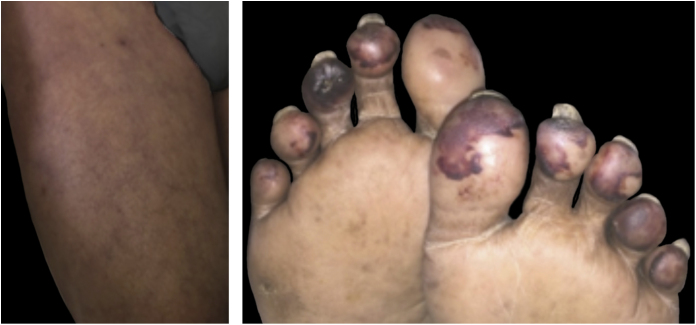
Livedo racemosa, purpura and necrosis of the extremities in a patient with COVID-19.

The cutaneous vascular manifestations associated with COVID-19 comprise a wide spectrum, ranging from transient to fixed livedo reticularis, livedo racemosa, purpura, and necrosis of the extremities (Fig. 3). They are rare manifestations associated with severe systemic conditions, which occur late in elderly patients, those hospitalized in an intensive care environment, with multiple comorbidities and laboratory alterations (for example coagulopathies, elevated D-dimer levels). They are frequently associated with other thrombotic events such as deep vein thrombosis, ischemic stroke (cerebrovascular accident) and disseminated intravascular coagulation, which is responsible for the high mortality in this group. Therefore, skin findings in patients with a potentially fatal disease are very specific and distinct from those found in the outpatient profile. This happens because probably the same microangiopathic and thrombotic mechanisms occur simultaneously in the main target organs, such as the lung and kidneys, and in the skin. Therefore, the treatment of skin lesions consists of treating the underlying disease itself.^16^, ^22^, ^26^ A summary of the main characteristics of the dermatological manifestations described above is found in Table 4 (Main characteristics of the skin manifestations associated with COVID-19).

**Table 4:** Main characteristics of skin manifestations associated with COVID-19.

	Dermatological picture	Frequency	Age range	Systemic picture	Lesion onset a
**Maculopapular eruption**	Exanthem, rash, squamous papular, pityriasis rosea-like, erythema multiforme-like	9%–47% 26, 27, 28, 29, 30	Adults	Mild	0–14 days
**Erythema Pernio-like**	Violaceous macules on the extremities at mild temperatures	18%–75% 26, 27, 28, 29, 30	Children and young individuals	Mild or asymptomatic	After 10–14 days
**Urticaria**	Indistinguishable from other forms of urticaria, angioedema is rare	9%–30% 26, 27, 28, 29, 30	Adults	Mild	Pre or 0–14 days
**Vesico-bullous**	Varicella-like	9%–15% 26, 27, 28, 29, 30	Adults	Mild to moderate	Pré or 0–7 days
**Livedo, purpura, necrosis**	Fixed livedo reticularis, livedo racemosa; purpura, vasculitis; necrosis of extremities	4%–9% 26, 27, 28, 29, 30	Elderly	Severe	After 10–14 days

a In relation to the onset of general symptoms of COVID-19.

## Other manifestations

### Mucosal involvement

Oral lesions during the course of COVID-19 have also been described and collected in review articles. It is known that SARS-CoV-2 can be detected in saliva using the PCR technique, which is currently used for diagnostic purposes. Moreover, ACE2, a functional viral receptor in the body, is present in the oral mucosa, with greater density on the dorsum of the tongue and minor salivary glands, which would justify the presence of mucosal lesions. Dysgeusia was the first oral symptom recognized in COVID-19, with a widely varying frequency, from five to 88%. Subsequently, numerous oral lesions were also described, with the most frequent being enanthema, petechiae, aphthoid lesions or ulcers, herpetiform lesions, candidiasis, and oral lesions associated with Kawasaki disease. They are usually symptomatic (pain, burning sensation or pruritus) and appear four days before or up to 12 weeks after the general symptoms. The treatment depends on the pattern of the lesion identified. Inadequate oral hygiene, immunosuppression, reactivation of latent viruses (for example, herpes virus), opportunistic infections, stress, trauma secondary to intubation, COVID-19 hyperinflammatory response are predisposing factors. Further studies are required to define which oral manifestations are directly linked to SARS-CoV-2 infection and which are indirectly due to the presence of other mucosal affections that, in these cases, occur in the context of COVID-19. In this sense, research is essential to exclude such conditions. It is suggested that vascular alterations and microthrombus-induced manifestations, such as petechiae and ulcers and enanthema (similar to viral exanthem) may be directly associated with the pathophysiology of SARS-CoV-2.^34^, ^35^, ^36^, ^37^, ^38^, ^39^, ^40^, ^41^, ^42^, ^43^, ^44^, ^45^, ^46^, ^47^, ^48^

### Telogen effluvium and COVID-19

Widely known in dermatology for a long time, telogen effluvium (TE) can be triggered by metabolic, nutritional changes, drug use, and several systemic conditions, including infections. In COVID-19, it is thought that the inflammatory cytokines released and the use of medications, such as heparin, may be involved in the TE mechanism. There is a report of an increase in the incidence of TE by almost threefold during the pandemic. It is estimated that 10% of patients with COVID-19 develop TE in the weeks and months following the infection, especially in patients with comorbidities, but it can occur even in subclinical conditions. To date, information about the onset, evolution, and prognosis of TE associated with COVID-19 are indistinguishable from TE due to other known causes. There is no peculiarity or specific characteristic in TE induced by SARS-CoV-2 infection. Therefore, the approach, treatment, and follow-up of these patients should be the same adopted for TE in dermatological practice.^49^, ^50^

### Case reports

In addition to the manifestations most often described and grouped into “patterns” in the large case series, prospective and systematic review studies, there are many isolated cases reports in the literature on several skin conditions possibly associated with COVID-19. Due to the methodological limitation inherent to case report-type publications, the actual role of SARS-CoV-2 infection in these entities cannot be affirmed, that is, whether they are in fact attributable to COVID-19 or not. The isolated conditions associated with COVID-19 that have been published in case reports are shown in Table 5 (Dermatological conditions associated with COVID-19 – Case reports).

**Table 5:** Dermatological conditions associated with COVID-19 - Case reports.

Urticarial	Vasculites and purpuras	Pharmacoderma-like	Specific topographies	Others
Urticaria	Leukocytoclastic vasculitis	Erythema multiforme	Ungual (red half-moon sign)	Pityriasis rosea-like
Urticarial vasculitis	IgA vasculitis	SDRIFE	Palmoplantar erythrodysesthesia	Grover’s disease
Angioedema	Eosinophilic granulomatosis with polyangiitis	TEN	Follicular eruption	Melkersson-Rosenthal syndrome
	Granulomatosis with polyangiitis	AGEP	Unilateral exanthem	Gianotti-Crosti syndrome
	Schamberg Purpura		Unilateral livedo	Erythema annulare centrifugum
	Purpura fulminans			Erythema nodosum
				Annular lichen planus
				Sarcoid lesion
				Granuloma annulare
				Alopecia areata

SDRIFE, dymmetrical drug related intertriginous and flexural exanthem; TEN, toxic epidermal necrolysis; AGEP, acute generalized exanthematous pustulosis.

The first reports were of cases of urticaria, which even preceded clinical symptoms,^51^ urticarial vasculitis,^52^ and angioedema.^53^ Also, cases of pityriasis rosea or pityriasis rosea-like lesions,^54^ with one report showing the presence of SARS-CoV-2 by immunohistochemical techniques ^55^ and another pointing to the possible role of HHV-6 reactivation and other viruses in these cases.^56^

There have also been reports of small-vessel vasculitis associated with COVID-19,^57^ leukocytoclastic vasculitis,^58^ IgA vasculitis,^59^ granulomatosis with polyangiitis (formerly called Wegener's disease),^60^ eosinophilic granulomatosis with polyangiitis (formerly called Churg-Strauss syndrome),^61^ Schamberg purpura^62^ and purpura fulminans.^63^

Regarding conditions that resemble drug reactions, patients with COVID-19 and erythema multiforme have been described,^64^ as well as case series of acute generalized exanthematous pustulosis (AGEP),^65^ reports of toxic epidermal necrolysis (TEN)^66^ and SDRIFE (symmetrical drug-related intertriginous and flexural exanthem).^67^

There are also case reports in specific topographies – ungual involvement, with a description of the “red half-moon sign”^68^; palmoplantar involvement with erythrodysesthesia^69^; follicular eruption,^70^ unilateral exanthem,^71^ unilateral livedo reticularis^72^ and pressure ulcers in unusual locations (such as the sternum, knees) from prolonged pronation.^73^ Dermoscopic descriptions of purpuric papular eruption^74^ and EPL lesions have been published.^75^

There are also reports of herpes zoster^76^ and isolated reports of Grover's disease-like conditions,^77^ Melkersson-Rosenthal syndrome,^78^ erythema nodosum,^79^ sarcoid lesion,^80^ Gianotti-Crosti syndrome,^81^ seborrheic dermatitis,^82^ annular lichen planus,^83^ erythema annulare centrifugum,^84^ granuloma annulare^85^ and rapidly progressive alopecia areata.^86^

## Histopathology of skin manifestations of COVID-19

Some studies presented the first descriptions of histopathological and immunohistochemical changes in skin lesions associated with COVID-19, aiming to contribute to the understanding of the pathophysiological mechanisms of these manifestations. The main histopathological changes of the most often described skin patterns will be briefly mentioned below.^87^

### Maculopapular eruptions

**Figure 4:**
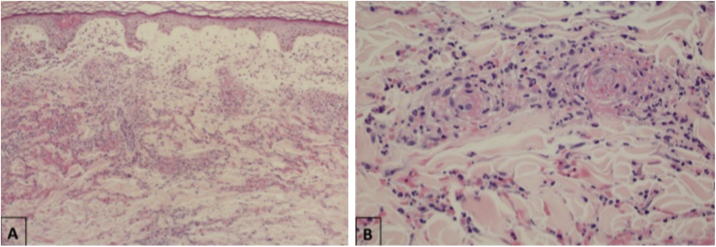
Histopathological findings of exanthem in a patient with COVID-19. (A), Leukocytoclastic vasculitis in the capillary vessels of the superficial dermis and erythrodiapedesis (Hematoxylin & eosin, ×100). (B), Capillary vessels containing intact and degenerated neutrophils partially destroying the wall, extravasated red blood cells, and the presence of eosinophilic amorphous material in the vascular lumen, suggestive of fibrin thrombus (Hematoxylin & eosin, ×400).

Superficial perivascular dermatitis with mild lymphocytosis, thrombosed vessels with neutrophilic and eosinophilic debris; interface dermatitis, superficial and deep perivascular lymphocytic infiltrate, with or without vasculitis (Fig. 4).

### Erythema pernio-like (EPL)

In the epidermis, presence of parakeratosis and hyperkeratosis, with apoptotic keratinocytes and, eventually, epidermal necrosis. Dense lymphocytic infiltrate in the superficial and deep dermis, also in the hypodermis, with a perivascular pattern and signs of endothelial activation. Sometimes, eccrine lymphocytic infiltrate is similar to that seen in eccrine lymphocytic hidradenitis. Other common findings are erythrodiapedesis, edema in the papillary dermis with the formation of a subepidermal bulla and increased interstitial mucin deposition. The presence of vascular microthrombi in the capillaries of the superficial dermis is rare, a well as in dermal venules.

### Urticarial eruptions

Perivascular lymphocytic infiltrate with some eosinophils and edema in the superficial dermis, eventually with vacuolar interface dermatitis and keratinocyte necrosis, with a pattern similar to that of erythema multiforme. In urticarial vasculitis, there is extravasation of red blood cells with perivascular neutrophilic infiltrate, macrophages, necrosis, and fibrin deposits.

### Vesicular eruptions

Acanthosis, vesicles due to vacuolar degeneration of the basal layer with dyskeratotic, multinucleated cells (suggestive of viral inclusion) without inflammatory infiltrate. In these cases, the differential diagnosis with herpetic dermatoviruses is essential.

### Livedo, purpura

Thrombotic vasculopathy with extensive deposition of C5b-9 and C4d (immunohistochemical technique) in the microvasculature.

### Androgens and COVID-19

Throughout the pandemic, SARS-CoV-2 infection was observed to affect more men than women. Men are twice as likely to be admitted to intensive care units. The association between the androgen pathway and SARS-CoV-2 infectivity has been described. Therefore, such predisposition to androgen-mediated infection could justify the high rates of hospitalization and mortality concentrated in men. Therefore, androgen expression in the form of alopecia (androgenetic alopecia – AGA) could, by itself, be a risk factor for COVID-19, and a high frequency of AGA has been reported among hospitalized men; and also the use of anti-androgens could be a protective and/or therapeutic factor in the evolution of these patients. In this sense, a published study showed that men with AGA or benign prostatic hyperplasia using a 5-alpha-reductase inhibitor who contracted COVID-19 showed a significant reduction in symptoms in the outpatient setting. In a case-control study in a hospital setting, men using 5-alpha-reductase inhibitors had a lower chance of being admitted to the ICU (Intensive Care Unit), suggesting this protective effect. However, it is still uncertain whether the introduction of anti-androgens after SARS-CoV-2 infection would provide any benefit. Apparently, dermatologists should encourage their AGA patients to maintain anti-androgen use during the pandemic. This is undoubtedly a very promising field of research.^88^, ^89^, ^90^

## Final considerations

Although the number of publications in a short period of time is significant and growing, there are still many challenging questions about the skin manifestations associated with COVID-19. The actual frequency of cutaneous findings is widely variable (0.2% to 45%) in the literature, and systematic review studies point to a frequency close to 6%. The most common dermatological manifestations were maculopapular eruptions, followed by EPL lesions. However, some publications mention that EPL lesions are the most prevalent manifestations. Urticarial, vesicular and livedo eruptions, in addition to purpura and necrosis, are less often described. As for the time of onset, skin lesions usually occur concomitantly with general symptoms and can rarely precede them, as in cases of urticaria and vesicular lesions. EPL can appear late or even be the only clinical manifestation of COVID-19. Vascular conditions such as livedo, purpura and necrosis are equally late manifestations but associated with greater severity and worse prognosis. Histopathological and immunohistochemical studies of the different patterns of skin lesions in patients with COVID-19 are essential for a better understanding of their pathophysiological significance in relation to the disease (are they directly caused by the viral infection? resulting from complications of systemic disease?).

It is worth emphasizing the importance of ruling out possible differential diagnoses in a patient with COVID-19 who presents skin lesions before attributing the skin condition exclusively to the viral infection. Spontaneous urticaria, other viral infections, drug reactions, among others, should be considered when investigating these patients.

The contribution of Dermatology to the COVID-19 pandemic seems to go beyond skin manifestations. The close relationship between androgens and the course of SARS-CoV-2 infection opens up a promising field of research on their prognostic and protective effects, especially regarding the systemic use of anti-androgens, widely prescribed in dermatology.

The pandemic has provided an excellent opportunity for dermatologists to learn and contribute. Unfortunately, it is not over yet. As science is a dynamic process, there is hope that more studies will be carried out so that answers can be found and uncertainties can be resolved.

## Financial support

None declared.

## Authors’ contributions

Camila Arai Seque: Design and planning of the study; analysis and interpretation of data; writing of the manuscript and critical review of the content; collection, analysis, and interpretation of data; critical review of the literature; approval of the final version of the manuscript.

Milvia Maria Simões and Silva Enokihara: Design and planning of the study; analysis and interpretation of data; writing of the manuscript and critical review of the content; collection, analysis, and interpretation of data; participation in research orientation; critical review of the literature; approval of the final version of the manuscript.

Adriana Maria Porro: Design and planning of the study; analysis and interpretation of data; writing of the manuscript and critical review of the content; collection, analysis, and interpretation of data; participation in research orientation; critical review of the literature; approval of the final version of the manuscript.

Jane Tomimori: Design and planning of the study; analysis and interpretation of data; statistical analysis; writing of the manuscript and critical review of the content; collection, analysis, and interpretation of data; participation in research orientation; critical review of the literature; approval of the final version of the manuscript.

## Conflicts of interest

None declared.
